# Analysis of prognostic factors of patients with malignant peritoneal mesothelioma

**DOI:** 10.1186/s12957-018-1350-5

**Published:** 2018-03-05

**Authors:** Wenjie Yin, Guoqi Zheng, Kunna Yang, Hui Song, Yufei Liang

**Affiliations:** 1 Department of Gastroenterology, Cangzhou Central Hospital, Cangzhou, Hebei 061001 China; 2 Department of Otolaryngology, Cangzhou Medical College, Cangzhou, Hebei 061001 China

**Keywords:** Malignant peritoneal mesothelioma, NLR, Albumin, Treatment, Prognostic factors

## Abstract

**Background:**

The study aims to find out independent prognostic factors for patients with malignant peritoneal mesothelioma (MPeM).

**Methods:**

Patients with pathologically proven MPeM were retrospectively reviewed. Potential prognostic factors were analyzed, including age, gender, asbestos exposure, body mass index (BMI), treatment, and laboratory results, such as blood routine examination and liver functions. The influences of various risk factors on the prognoses were analyzed by univariate analysis. A Cox regression model analysis established independent factors for the survival prognosis of the patients.

**Results:**

Seventy MPeM patients, including 33 patients who received intraperitoneal chemotherapy with cisplatin, 14 patients who received systemic chemotherapy with cisplatin + pemetrexed, and 21 untreated patients were included in this study. The 1-year survival was 32.9%, the 2-year survival was 10%, and the 3-year survival was 2.9%. The median age of MPeM was 62 years, and the female-to-male ratio was 1:0.56. The univariate and multivariate analyses showed that treatment, albumin (ALB), and blood neutrophil-to-lymphocyte ratio (NLR) were independent factors that affected the overall survival (OS) of MPeM patients.

**Conclusion:**

High blood NLR and hypoalbuminemia are adverse prognostic factors for MPeM patients. Systemic chemotherapy and intraperitoneal chemotherapy can prolong the survival period.

## Background

Malignant peritoneal mesothelioma (MPeM) is a mesothelium-derived neoplasm with high malignancy that is commonly caused by exposure to asbestos [1]. MPeM is a very rare disease, the incidence was 1–2 per million [2]. In Cangzhou, Hebei Province, China, the incidence of MPeM is approximately 4.5 per million, possibly because asbestos was widely used in the 1970s in this region and sanitation was poor [3].

MPeM has a poor prognosis. Most patients die from the disease within 1 year. Therefore, we tried to identify the prognostic factors of MPeM to direct future clinical medication.

Currently, the pathogenesis is unclear. The pathogenesis of malignant mesothelioma (MM) is associated with chronic inflammation [4]. In recent years, researchers have been interested in systemic inflammation. Since 1863, when the German pathologist Rudolf Vichow found white blood cells in tumor tissue, several hypotheses based on the results of research have been advanced proposing that neoplasms occur at inflammation sites. The connection between the tumor and inflammation can be reflected in a series of indexes of blood parameters. The blood neutrophil-to-lymphocyte ratio (NLR) is systemic markers of inflammation. Strong evidence suggests that the NLR can be used to indicate the inflammation condition. Previous studies have shown that NLR is an independent prognostic factor for many cancers, such as colorectal cancer, breast cancer, soft tissue sarcoma, and bladder cancer [5–9].

Nutritional status affects the outcomes of patients with malignant cancers, including therapeutic interventions, the length of hospitalization, and the prognosis [10–12]. Serum albumin (ALB) can indicate the nutritional status, which is an independent prognostic factor for many cancers, including pancreatic carcinoma, gastric carcinoma, nasopharyngeal carcinoma, bladder cancer, and malignant pleural mesothelioma [13–17].

The immunological status and nutritional condition are important prognostic factors for patients with malignant cancers. However, no study has confirmed the relationship between NLR and ALB and survival in MPeM patients. Therefore, the aim of this study was to determine whether NLR and ALB could predict OS in MPeM patients. This article also discussed the possible mechanism.

## Methods

### Patients

From January 2010 to December 2014, 70 consecutive patients with pathologically proven MPeM in Cangzhou Central Hospital were retrospectively reviewed. The MPeM diagnosis was made according to the Guidelines for Pathologic Diagnosis of Malignant Mesothelioma: 2012 Update of the Consensus Statement from the International Mesothelioma Interest Group [18]. All patients underwent ultrasound guided percutaneous peritoneal puncture biopsy. The data were collected before treatment. Cytoreductive surgery and perioperative intraperitoneal chemotherapy are rarely applied in our region. The statistics referenced for survival with malignant peritoneal mesothelioma refer to patients who do not undergo aggressive cytoreductive surgery.

### Baseline variables

Patient characteristics and laboratory results were examined as potential prognostic factors, including age, gender, treatment, asbestos exposure, body mass index (BMI), neutrophil count, platelet count, NLR, and ALB. According to previous publications and the actual situation, the factors were categorized as follows: NLR, < 3 versus ≥ 3 [19]; ALB, < 35 g/l versus ≥ 35 g/l [13]; platelet count, ≥ 338 × 10^9^/l versus < 338 × 10^9^/l; WBC count, > 6.23 × 10^9^/l versus < 6.23 × 10^9^/l; age, < 63 years versus ≥ 63 years [4]; gender, male versus female; and BMI, < 22.79 versus ≥ 22.79.

### Statistical analysis

Survival was estimated using the Kaplan–Meier method. Potential prognosticators were submitted to univariate and multivariate analyses. The Kaplan–Meier model was used to compare the survival rate among groups. Multivariate Cox proportional hazards models were used to identify factors linked to the prognosis. These variables include age, NLR, ALB, gender, asbestos exposure, and treatment. The Cox regression modeling results are presented as hazard ratios (HR) with associated 95% confidence intervals (CIs). A difference with a *p* value less than 0.05 was considered statistically significant. All data were analyzed with the system of SPSS 22.0.

## Results

### Patient characteristics

**Fig. 1:**
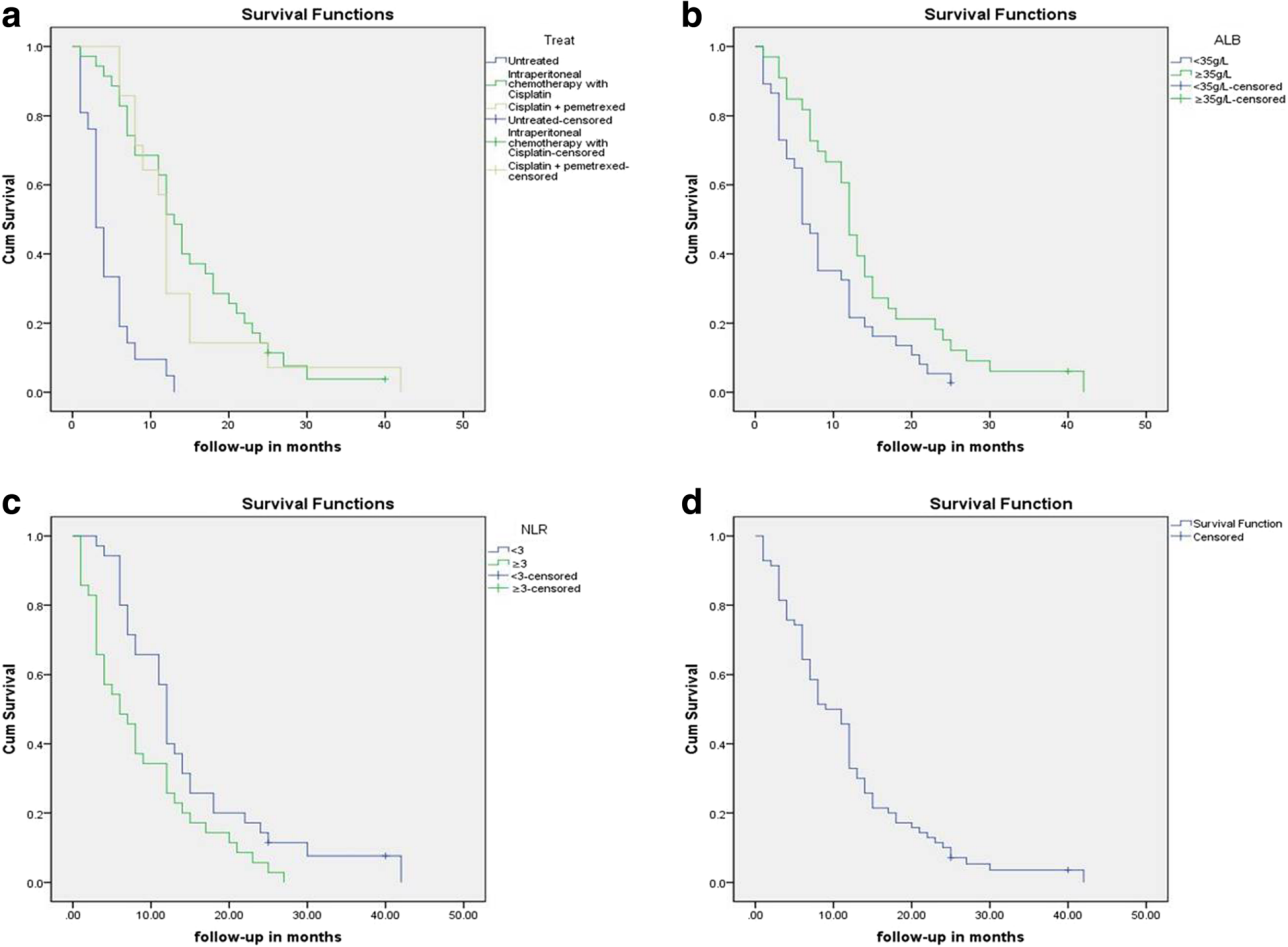
**a** The survival function of the original lifetime data. **b** Kaplan–Meier survival curves depicting OS according to the ALB. The OS rate of patients with ALB < 35 g/l was significantly lower than that of patients with ALB ≥ 35 g/l (*P* = 0.018). **c** Kaplan–Meier survival curves depicting OS according to the NLR. The OS rate of patients with a NLR < 3 was significantly higher than that of patients with a NLR ≥ 3 (*P* = 0.012). **d** Kaplan–Meier survival curves depicting OS according to the treatment. The OS rate of untreated patients was significantly lower than that of treated patients (*P* = 0.000). There was no significant difference between the patients receiving intraperitoneal chemotherapy with cisplatin and the patients receiving systemic chemotherapy with cisplatin + pemetrexed (*P* > 0.05)

A total of 70 MPeM patients, including 35 patients receiving intraperitoneal chemotherapy with Cisplatin, 14 patients receiving systemic chemotherapy with cisplatin + pemetrexed, and 21 untreated patients, were included in this retrospective evaluation (Table 1). As of the date of this report, the median OS was 10 months (range 1–42 months). The 1-year survival was 45.8%, the 2-year survival was 11.4%, and the 3-year survival was 2.9% (Fig. 1a). The median age of the patients was 62 years (42–85). In our study, the female to male ratio is 1:0.56. 57 (81.4%) patients had a history of asbestos exposure in the MPeM patients. Abdominal distension (71.4%) and abdominal pain (38.7%) were the most frequent manifestations. The median NLR was 3 (SD = 2.47). A total of 35 patients (50%) had a NLR ≥ 3. The median ALB level was 34 g/L (SD = 5.244). Thirty-seven patients (52.9%) had hypoalbuminemia (ALB < 35 g/L).

**Table 1:** Patient characteristics (*n* = 70)

Characteristics	Number of patients
Median age (years)	62 (42–85)
Sex (male/female)	25/45 (36%/64%)
Asbestos exposure	57 (81.4%)
Mesian BMI	22.79 (15.23–31.49)
Median survival (months)	10 (1–42)
Treatment	
Intraperitoneal chemotherapy with cisplatin	35 (50%)
Cisplatin + pemetrexed	14 (20%)
Untreated	21 (30%)
Symptoms	
abdominal distension	50 (71.4%)
abdominal pain	25 (35.8%)
Histology type	
Epithelioid	50 (71.4%)
Biphasic + sarcomatoid	20 (28.6%)
NLR (range)	0.69–18.46
NLR ≥ 3	35 (50%)
NLR < 3	35 (50%)
ALB (range)	23–48 g/L
Normal albumin (ALB ≥ 35 g/L)	33 (47.1%)
Hypoalbuminemia (ALB < 35 g/L)	37 (52.9%)

### Univariate and multivariate analysis for prognostic factors

The prediction factors of OS were determined by Kaplan–Meier model in univariate analysis. In the univariate analysis, NLR, ALB, and treatment were related to the prognosis of patients with MPeM. When these variables were added to a multivariate Cox model, ALB, NLR, and treatment were independent prognostic factors (Table 2). Patients were divided into high NLR (NLR ≥ 3) group and low NLR (NLR < 3) group, high ALB (ALB ≥ 35 g/L) and low ALB (ALB< 35 g/L) group. The Kaplan–Meier method and log-rank test indicated that ALB ≥ 35 g/L and NLR < 3 were associated with longer OS (Fig. 1b, c).

**Table 2:** Univariate and multivariate analysis for prognostic factors

	Univariate analysis			Multivariate analysis		
	HR	95% CI	*P*	HR	95% CI	*P*
Age (years)	1.012	0.983–1.041	0.431	0.993	0.963–1.025	0.672
Gender (male/female)	0.747	0.448–1.245	0.263	0.959	0.549–1.674	0.882
BMI	0.949	0.891–1.011	0.105			
Asbestos exposure (yes/no)	1.082	0.588–1.992	0.8	1.135	0.636–2.025	0.669
Histological subtype (Epithelial/biphasic + sarcomatoid)	0.800	0.468–1.367	0.415			
Treatment Cisplatin Cisplatin + pemetrexed Untreated	0.418	0.263–0.664	0.00	0.475	0.289–0.782	0.003
WBC count	0.966	0.829–1.126	0.661			
PLT count	1.001	0.998–1.004	0.461			
NLR	1.176	1.062–1.303	0.002	1.138	1.015–1.276	0.027
ALB	0.920	0.872–0.971	0.002	0.935	0.879–0.994	0.031

### ALB and survival

Median survival at the end of follow-up was 12 months in patients with ALB ≥ 35 g/L. The median survival of patients with hypoalbuminemia (ALB < 35 g/L) was 6 months. The 1-year survival rate was 32.4% for patients with ALB < 35 g/L and 60.6% for patients with ALB ≥ 35 g/L (Fig. 1b).

The two groups (ALB ≥ 35 g/L and < 35 g/L) were analyzed regarding other prognostic indicators for MPeM. Statistical analysis was performed with the chi-square test. The results showed ALB was concerned with age (*P* < 0.05), but was not concerned with gender, BMI, asbestos exposure, treatment, and histological subtype (*P* > 0.05; Table 3).

**Table 3:** Prognostic factors for MPeM stratified according to ALB levels (χ^2^ test)

Variable	Number of patients stratified by ALB		*P* value
	Hypoalbuminemia (ALB < 35 g/l)	Normal albumin (ALB ≥ 35 g/l)	
Age (years)			
< 63	19	25	
≥ 63	18	8	0.01 < *P* < 0.05
Gender			
Male	13	12	
Female	24	21	*P* > 0.05
BMI			
< 22.79	23	17	
≥ 22.79	14	16	*P* > 0.05
Asbestos exposure			
Yes	31	26	
No	6	7	*P* > 0.05
Treatment			
Cisplatin	17	18	
Cisplatin + pemetrexed	6	8	
Untreated	14	7	*P* > 0.05
Histological subtype			
Epithelial	28	22	
Biphasic + sarcomatoid	9	11	*P* > 0.05

### NLR and survival

Median survival at the end of follow-up was 12 months in patients with NLR < 3. The median survival of patients with NLR ≥ 3 was 6 months. The 1-year survival rate was 32.4% for patients with NLR ≥ 3 and 57.1% for patients with NLR < 3 (Fig. 1c).

The two groups (NLR < 3 and NLR ≥ 3) were analyzed regarding other prognostic indicators for MPeM. Statistical analysis was performed with the chi-square test. There was no significant correlation between NLR and age, gender, BMI, asbestos exposure, treatment, and histological subtype (*P* > 0.05; Table 4).

**Table 4:** Prognostic factors for MPeM stratified according to NLR levels (χ^2^ test)

Variable	Number of patients stratified by NLR		*P* value
	NLR < 3	NLR ≥ 3	
Age (years)			
< 63	25	19	
≥ 63	10	16	*P* > 0.05
Gender			
Male	10	15	
Female	25	20	*P* > 0.05
BMI			
< 22.79	17	18	
≥ 22.79	18	17	*P* > 0.05
Asbestos exposure			
Yes	29	28	
No	6	7	*P* > 0.05
Treatment			
Cisplatin	20	15	
Cisplatin + pemetrexed	8	6	
Untreated	7	14	*P* > 0.05
Histological subtype			
Epithelial	24	26	
Biphasic + sarcomatoid	11	9	*P* > 0.05

### Treatment and survival

The Kaplan–Meier curve for OS was stratified by treatment. The result indicated that non-treatment was associated with shorter OS (*P* = 0.000; Fig. 1d). The 1-year survival rate was 62.9% for patients receiving intraperitoneal chemotherapy with cisplatin, 57.1% for patients receiving systemic chemotherapy with cisplatin + pemetrexed, and 9.5% for untreated patients (Fig. 1d). The mean survival was approximately 14.6 months for the cisplatin group, 13.8 months for the cisplatin + pemetrexed group and 4.5 months for the untreated group. There was no significant difference between the patients receiving intraperitoneal chemotherapy with cisplatin and the patients receiving systemic chemotherapy with cisplatin + pemetrexed (*P* > 0.05).

The three groups were analyzed regarding other prognostic indicators for MPeM. Statistical analysis was performed with the chi-square test. The three groups had differences in age (*P* < 0.05) but did not show any significant differences with gender, BMI, asbestos exposure, and histological subtype (*P* > 0.05; Table 5).

**Table 5:** Prognostic factors for MPeM stratified according to treatment (χ^2^ test)

Variable	Treatment			*P* value
	Cisplatin	Cisplatin + pemetrexed	Non-treatment	
Age (years)				
< 63	21	10	6	
≥ 63	15	4	15	*P* < 0.05
Gender				
Male	10	5	10	
Female	25	9	11	*P* > 0.05
BMI				
< 22.79	15	7	13	
≥ 22.79	20	7	8	*P* > 0.05
Asbestos exposure				
Yes	28	13	16	
No	7	1	5	*P* > 0.05
Histological subtype				
Epithelial	23	10	17	
Biphasic + sarcomatoid	12	4	4	*P* > 0.05

## Discussion

MPeM is a mesothelium-derived carcinoma with high malignancy. For most tumors, depth of tumor invasion, tumor differentiation, the number of lymph nodes metastatic field, and tumor location were of prognostic significance. MPeM exhibits local aggressiveness, but only rare distant metastases [20–22]. Unlike other solid tumors, currently, there is some controversy on how best to assign the tumor grade in MPeM [19].

Up until now, there is no systematic assessment of prognosis in peritoneal mesothelioma. Therefore, the effective identification of MPeM prognostic factors will play an important role in clinical management. A few articles reported some prognostic factors of patients with malignant cancers, including age, gender, asbestos exposure, lymph node metastases, estrogen receptors, mesothelin, GLUT1, morphological growth patterns, and the mitotic index [23–28]. The current study found that the simple laboratory indicators NRL and ALB could predict OS in MPeM patients.

Most patients with MPeM have a history of asbestos exposure. The asbestos fibers are thought to skewer cells and set off chemical reactions that lead to inflammation, DNA damage, and cell death. Inflammation is critical during tumor initiation and malignant progression. Recently, many people have focused on the role of inflammation in cancer. Peripheral blood leukocyte counts can reflect and detect the degree of the systemic inflammatory response in tumor patients, which is a simple and valuable indicator [29]. NLR, a systemic marker for inflammation, have been found to predict the prognosis of tumor patients.

The specific mechanism by which the NLR affects the prognosis of patients with tumors is not clear. The cellular immunity induced by lymphocytes plays a very important role in the anti-tumor process. Peripheral blood lymphocytes are decreased in patients with a high NLR, and the antitumor response is reduced. This phenomenon provides an appropriate growth environment for cancer cells, thereby enabling their proliferation and metastasis. In contrast, neutrophils are the major source of vascular endothelial growth factor (VEGF) production. VEGF expression in tumors influences the formation of tumor vessels. Tumor-associated angiogenesis plays a pivotal role in tumor growth and metastasis. However, a high NLR is not simply an imbalance in the lymphocyte and neutrophil counts. Tumor cells secrete myeloid growth factors, which induce leukocyte proliferation [30]. Thus, the immune mechanism is very complex.

The NLR can be easily calculated from differential WBC counts obtained through routine procedures. There is ample evidence indicating the role of neutrophils in cancer pathophysiology. The NLR is closely related to the mortality rate and the response to treatment, and the NLR can predict the prognosis [5–9]. Patients have been stratified according to theirs NLRs, but non-conformity exists in the layer boundary points. Cihan et al. [30] and Kao et al. [19] confirmed 3 as a dividing point, Kao et al. [4] confirmed 5, and Shen et al. [31] confirmed 2.8. In our study, the median NLR was 3; therefore, we confirmed 3 as a dividing point. The Kaplan–Meier curve for OS was stratified by the NLR. The median OS was 12 months versus 6 months for a NLR < 3 versus ≥ 3, respectively. The Kaplan–Meier method and the log-rank test indicated that a NLR < 3 was associated with longer OS (*P* = 0.012; Fig. 1c). The 1-year survival rate was 32.4% for patients with a NLR ≥ 3 and 57.1% for patients with a NLR < 3 (Fig. 1c). We compared interclass equilibration involving age, gender, BMI, asbestos exposure, treatment, and histological subtype (Table 4) but found no significant differences (*P* > 0.05). The Cox proportional regression analysis showed that a NLR ≥ 3 was an independent adverse prognostic factor for MPeM.

Cancer is a cause of malnutrition. Malnutrition plays an important role in the short OS, decreased quality of life, and increased mortality of malignant tumors [10–12]. The serum albumin level is the most commonly used serological indicator to evaluate malnutrition. Several studies have confirmed that serum albumin, which is a simple and objective indicator of the nutritional status, is an independent prognostic factor for several cancers, including malignant pleural mesothelioma [13], pancreatic carcinoma [14], gastric carcinoma [15], nasopharyngeal carcinoma [16], and bladder cancer [17].

Serum albumin synthesis in the liver is an important in vivo physiological function of macromolecules. Albumin can maintain stable plasma colloid osmotic pressure and enhance immune functions and has some anti-tumor effects. Total serum protein and albumin reflect the body’s absorption, synthesis and decomposition of proteins, and the albumin content reflects the immune response to some extent [32]. Most carcinoma patients have hypoalbuminemia. Hypoalbuminemia is not only a result of an insufficient or poorly balanced diet, faulty digestion, or utilization of foods but also occurs because tumor cells consume a large amount of nutrients to grow. In our study, 37 patients (52.9%) had hypoalbuminemia (ALB < 35 g/L) and 33 patients (47.1%) had an ALB level ≥ 35 g/L (Table 1). The Kaplan–Meier method and the log-rank test indicated that a normal albumin level was associated with longer OS (*P* = 0.018; Fig. 1b). ALB was associated with age (*P* < 0.05) but did not show any significant association with gender, BMI, asbestos exposure, treatment, and histological subtype (*P* > 0.05; Table 3). The Cox proportional regression analysis showed that hypoalbuminemia was an independent adverse prognostic factor for MPeM.

NLR and ALB are simple, inexpensive, and commonly performed laboratory tests. Blood cell analysis and hepatic functions are routine exams. ALB is measured as a part of the hepatic function tests, and the NLR is defined as the absolute neutrophil count divided by the absolute lymphocyte count. Therefore, I stress that the potential prognostic roles of ALB and NLR in MPeM are important.

The main treatment methods for MPeM are cytoreductive surgery combined with adjuvant chemotherapy, systemic chemotherapy, and intraperitoneal chemotherapy. There is no standard of therapy for MPeM. The optimal treatment of MPeM remains controversial. Pemetrexed combined with cisplatin has been approved as a first-line therapy for MPeM [33, 34]. Jänne et al. reported a median survival of 13 months in 66 MPeM patients treated with systemic pemetrexed and cisplatin versus 9 months for 32 diffuse malignant peritoneal mesothelioma (DMPM) patients treated with systemic pemetrexed alone [35]. Cisplatin was one of the first chemotherapy drugs used in intraperitoneal chemotherapy. Intraperitoneal chemotherapy has the following merits: high concentration of cisplatin in the peritoneal cavity, higher local tumoricidal effect, lower nephric toxicity, and lower systemic toxicity [36]. Recently, several prospective trials have confirmed a median OS of 40 to 90 months and a 5-year survival of 30 to 60% after combined treatment using cytoreductive surgery and perioperative intraperitoneal chemotherapy [37]. The Washington Cancer Institute (Washington DC, USA) recently published an updated series on 100 MPeM patients who underwent combined treatment and demonstrated that the median OS was 52 months, with a 5-year survival of 46% [38]. Cytoreductive surgery and perioperative intraperitoneal chemotherapy are rarely applied in our region. None of the patients undergo aggressive cytoreductive surgery in this group. Similar to the previous report, in our study, the mean survival was approximately 14.6 months for the cisplatin group, 13.8 months for the cisplatin + pemetrexed group and 4.5 months for the non-treatment group. Systemic chemotherapy (cisplatin + pemetrexed) and intraperitoneal chemotherapy (cisplatin) both obviously prolong the survival period (*P* < 0.05), with no significant difference between them (*P* > 0.05).

Previous studies have shown that gender, asbestos exposure, and histological subtype are associated with OS in MPeM [21, 23, 39]. However, in our study, none of above-mentioned factors was shown to be a predictor of MPeM. The explanation for this finding may be that the research object in our study is different. Contrary to earlier reports, more men suffer from MPeM than women. However, the incidence was higher in females than in males in Cangzhou, Hebei Province, China. In the 1970s, it was women who were involved in large handspun asbestos processes in this area. The exposure time and intensities were higher in females than in males [17]. In our study, 25 patients (36%) were male and 45 patients were female (64%). In the MPeM group, 57 patients (81.4%) had a history of asbestos exposure. The median age at diagnosis for the patients was 62 years (range 42–85 years).

The limitations of our study were that the clinical examination was primarily performed based on retrospective observations. So, we need a larger prospective study to verify the results.

## Conclusion

In conclusion, the study showed that a high blood NLR and hypoalbuminemia were adverse prognostic factors for MPeM patients. Systemic chemotherapy and intraperitoneal chemotherapy obviously prolonged the survival period.
